# Targeting Metal Imbalance and Oxidative Stress in Alzheimer’s Disease with Novel Multifunctional Compounds

**DOI:** 10.3390/molecules30173512

**Published:** 2025-08-27

**Authors:** Eleftherios Charissopoulos, Eleni Pontiki

**Affiliations:** Laboratory of Pharmaceutical Chemistry, School of Pharmacy, Faculty of Health Sciences, Aristotle University of Thessaloniki, 54124 Thessaloniki, Greece; echariss@pharm.auth.gr

**Keywords:** Alzheimer’s disease, amyloid-β, metal chelators, antioxidants

## Abstract

Alzheimer’s disease (AD) is considered to be one of the most common types of dementia, threatening the health of elderly individuals. Enhancing the brain’s cholinergic activity is currently the primary therapeutic strategy for treating AD patients. Acetylcholine and butyrylcholine are key targets in this approach, as they function as neuromodulators within the cerebrum—particularly in its various cholinergic regions responsible for essential functions like memory, thought, inspiration, and excitement. Oxidative stress and free radicals are considered to play a crucial role in the pathogenesis of AD and may be key factors in its etiology. Additionally, oxidants and oxidative stress-induced products can upregulate amyloid precursor protein (APP) expression, promoting Aβ aggregation. Another major factor in the pathogenesis of AD is the imbalance of metal homeostasis in the brain. Notably, the mammalian brain contains significantly higher concentrations of Cu, Zn, and Fe ions compared to other tissues. The present review focuses on novel bifunctional metal chelators with potential antioxidant activity for the treatment of AD.

## 1. Introduction

Alzheimer’s disease (AD) is a heterogeneous disease with a complex pathobiology, accounting for 60–70% of dementia cases, occurring after the age of 65 [1,2]. AD is the leading cause of dementia, often beginning with mild and easily overlooked memory impairment that progressively worsens over time and is one of the most expensive, deadly, and burdensome diseases of our century [3,4]. AD is neuropathologically marked by the presence of senile plaques and neurofibrillary tangles (NFTs) [5]. AD is broadly categorized into two principal types: early-onset AD (EOAD) and sporadic or late-onset AD (LOAD), with LOAD accounting for over 95% of all cases. An abnormal accumulation of the toxic form of amyloid-β (Aβ) is observed in both types, resulting from an imbalance between its production and clearance [2,6,7]. Pathologically, there is severe neuronal loss, formation of intraneuronal neurofibrillary tangles composed of hyperphosphorylated tau protein, and aggregation of Aβ in extracellular senile plaques [8,9]. These characteristics are recognied as the primary neuropathological criteria for the diagnosis of AD [10]. The blood–brain barrier (BBB) protects the central nervous system (CNS) from toxins and pathogens in the blood. The BBB prevents most chemical drugs and biopharmaceuticals from entering the brain, making it hard to treat CNS disorders. The barrier regulates the entrance and expulsion of molecules from the vascular compartment to the brain in conjunction with a group of receptors, transporters, efflux pumps, and other cellular components [11]. Selective BBB and neurovascular coupling are two unique central nervous system vasculature features. In AD, vascular dysfunction and BBB disruption are observed, mediated by various molecular and genetic alterations [12].

### 1.1. Amyloid Plaques

In the brain, there is high expression of a single-pass transmembrane protein called APP. APP is rapidly metabolized through a complex process [13,14]. Alpha- and gamma-secretases are responsible for the cleavage of APP that leads to the production of insoluble Aβ peptides [15,16,17]. Aβ, initially discovered in senile plaques, was originally believed to be an abnormal protein [18]. In a healthy brain, APP is initially cleaved by β-secretase, generating soluble APP fragments. Subsequently, γ-secretase cleaves the remaining membrane-bound portion, producing peptides that are released extracellularly and are rapidly degraded or cleared [15,19]. Current studies suggest that Aβ—whether in the form of plaques or as non-fibrillar, soluble, oligomeric forms—initiates a pathophysiological cascade that results in tau misfolding and aggregation, which subsequently propagates throughout the cortex [20,21]. The toxic effects of Aβ may involve synaptic impairment, excitotoxic damage, changes in membrane permeability, disruption of calcium balance, inflammation, and oxidative stress [22,23].

### 1.2. Neurofibrillary Tangles (NFTs)

NFTs represent a core neuropathological feature of AD [24]. NFTs are composed of abnormally phosphorylated forms of the microtubule-associated protein tau [25]. The tau filaments observed in AD have been termed “paired helical filaments” (PHFs). In AD, tau proteins undergo hyperphosphorylation and abnormal folding, which reduce their ability to bind to and stabilize microtubules in the axon. The loss of normal tau function, combined with the enhanced accumulation of abnormal tau, facilitates the aggregation of PHFs with normal tau proteins [26]. When NFTs occur in the absence of Aβ plaques, the condition is termed primary age-related tauopathy (PART). When Aβ plaques are also present, NFT pathology is currently recognized as AD neuropathological change (ADNC) [27]. Synapse loss and the formation of NFTs have been shown to be strongly correlated throughout the progression of AD. Both factors play a crucial role in cognitive decline, suggesting that these AD lesions occur in a related, rather than independent, manner [24].

### 1.3. Synaptic Loss

Synaptic loss is regarded as a key pathological feature contributing to the progression of AD. Several studies have reported that cognitive dysfunction is associated with a loss of dendritic spines. The shape of spines is primarily determined by the cytoskeletal protein F-actin (fibrillar actin), and exposure to Aβ can trigger F-actin depolymerization into G-actin (globular actin), leading to spine collapse [28]. Synaptic proteins, such as neurogranin, a postsynaptic neuronal protein, visinin-like protein-1 (VILIP-1), and synaptotagmin-1 serve as biomarkers for the detection and quantification of synaptic loss and its severity [29].

### 1.4. Oxidative Stress and Alzheimer’s Disease

A significant imbalance between the production of reactive oxygen species (ROS) and reactive nitrogen species (RNS) is known as oxidative stress [30,31,32]. Evidence suggests that oxidative stress and free radicals are involved in pathogenesis and may play a role in the etiology of Alzheimer’s disease (AD). This results in the damage of essential cellular components, including nucleic acids, lipids and proteins [33,34,35,36]. Indeed, it has been shown that mitochondrial dysfunction, plays a significant role in the pathophysiology of AD, through the generation of reactive oxygen species (ROS) [37]. Oxidative stress has been shown to play a pivotal role in the modification of APP and the regulation of secretase enzyme activities. Oxidants and oxidative products produced through oxidative stress can affect the expression of APP by increasing it, which in turn promotes the aggregation of Aβ [38]. Concurrently, peptide Aβ and the presence of trace metal ions, such as iron and copper, have been identified as potential sources of oxidative stress. The insertion of Aβ oligomers into the lipid bilayer can lead to the production of reactive oxygen species (ROS) [37].

### 1.5. Metal Dyshomeostasis in Alzheimer’s Disease

An imbalance of metal homeostasis in the brain is one of the most crucial factors in the pathogenesis of AD. Significantly high concentrations of Cu, Zn, and Fe ions are detected in the mammalian brain in comparison to other tissues [39,40,41,42]. The concentrations of Cu, Zn, and Fe in the brain are tightly regulated at the level of the BBB [40]. Furthermore, oxidative stress is closely linked to the imbalance of metal homeostasis in the brain [41]. It has been reported that restoring proper metal ion balance in the brain can halt Aβ aggregation, disassemble amyloid plaques, and slow AD-related cognitive decline in both patients with AD and AD transgenic mouse models [41,43]. Copper and zinc play crucial roles in brain function. Copper is a vital trace element involved in energy production, neurotransmission, and free radical scavenging. Zinc is also an essential trace element that participates in neurotransmission and redox regulation. Aβ plaques exhibit a high binding affinity for the trace metals copper and zinc and have been found to contain elevated concentrations of these metals [44]. Iron, an essential trace element, serves as a cofactor for numerous physiological processes in the brain, including oxygen transport, mitochondrial respiration, DNA synthesis, and phospholipid synthesis [45].

### 1.6. Metal Chelators as Potential Drugs Against AD

**Figure 1:**
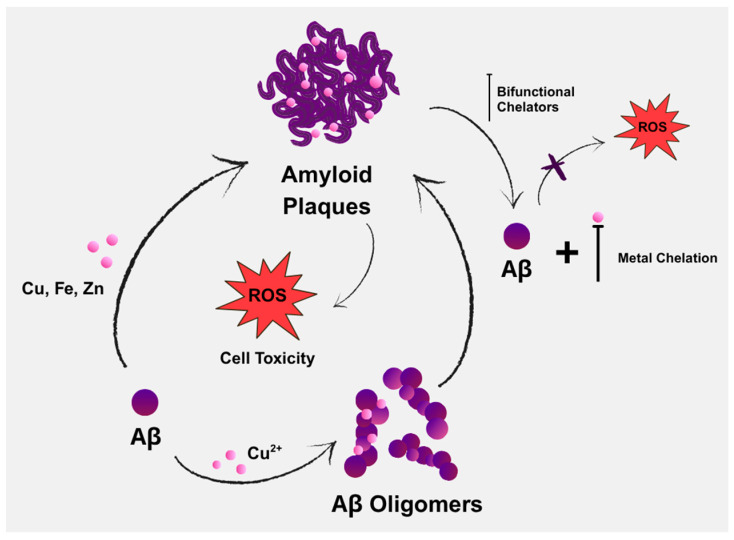
Contribution of endogenous metals in the aggregation of amyloid-beta (Aβ), along with the potential role of bifunctional chelators in AD therapy through chelation.

Aβ deposition and oxidative stress are two of the main pathognomonic features of AD (Figure 1), both of which are driven by Aβ’s interactions with metal ions [46]. Copper is considered a crucial metal in the development of chelators for treating AD. It not only shows the most pronounced dyshomeostasis in amyloid plaques, but also significantly affects Aβ homeostasis by increasing Aβ deposition and contributing to neuroinflammation. Another possible risk is zinc dyshomeostasis, due to extracellular overload within the amyloid plaques or intracellular overload/deficit [47].

## 2. Novel Bifuctional Metal Chelators

### 2.1. Zinc Metal Carboxylates

**Figure 2:**
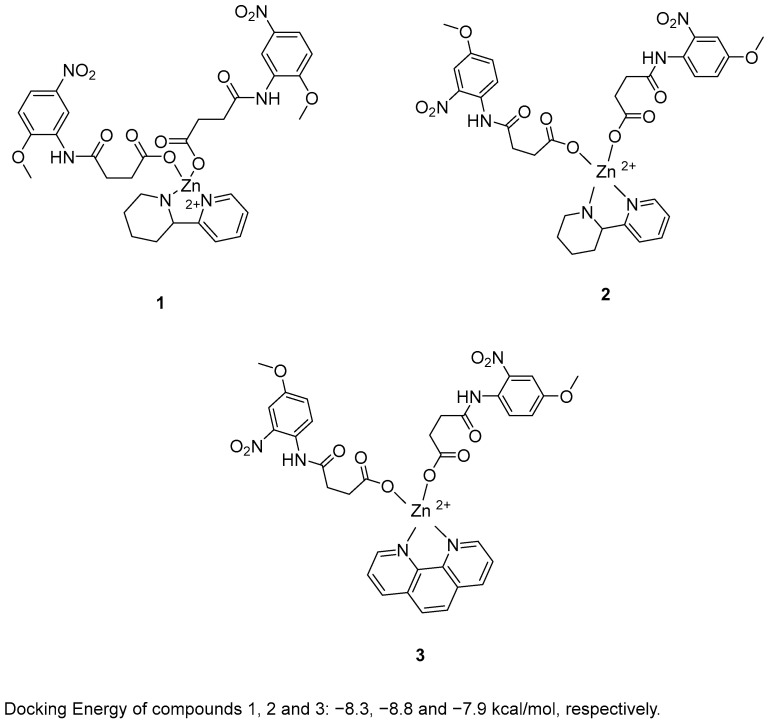
Chemical structures of zinc metal carboxylates (**1**–**3**) [48].

Zafar, R. et al. [48] synthesized and biologically evaluated novel zinc metal carboxylates. All synthesized compounds were evaluated for their inhibitory activity against acetylcholinesterase (AChE) and butyrylcholinesterase (BChE) using Ellman’s assay. Compounds **1** and **2** exhibited promising AChE inhibitory activity, with IC_50_ values of 59.52 and 33.07 μM, respectively, compared to the reference compound galantamine (IC_50_ = 1.67 μM). In the BChE assay, compound **3** demonstrated remarkable inhibitory activity, with an IC_50_ value of 0.056 μM. Galantamine was used as the reference compound, exhibiting an IC_50_ of 2.13 μM. Despite its extraordinary results in the BChE assay, compound **3** exhibited weak AChE inhibition. In the BChE assay, compound **3** exhibited activity comparable to its performance in the AChE assay, with an IC_50_ value of 55.56 μM. To evaluate their antioxidant potential, the DPPH radical scavenging activity of all six synthesized compounds was assessed. Ascorbic acid was used as a standard with IC_50_ = 3.62 µM. Compounds **1**, **2** and **3** (Figure 2) were proved to be strong antioxidants with IC_50_ values of 0.927, 0.245 and 0.401 µM. In addition, the ABTS assay revealed that the compounds possessed significantly elevated antioxidant activity. In particular, compound **2** exhibited the strongest antioxidant activity, with an IC_50_ value of 0.070 µM. Compounds **1** and **3** also showed notable activity, with IC_50_ values of 1.15 and 0.721 µM, respectively. Ascorbic acid was used as the reference compound (IC_50_ = 2.03 µM). Molecular docking analysis of compound **5** was performed at the active site of the crystal structure of acetyl cholinesterase AChE enzyme. The results showed that compounds **1**, **2** and **3** binds to the enzyme with docking energy of −8.3, −8.8 and −7.9 kcal/mol, respectively. Compound **2** exhibited the best results in all assays except for BChE inhibition. In terms of AChE inhibition, the 2-nitro and 4-methoxy substitutions on the aromatic ring, as present in compound **2** (IC_50_ = 33.07 µM), were more favorable compared to compound **1** (IC_50_ = 141.02 µM). This was also observed in the DPPH and ABTS assays. The presence of 2-(piperidin-2-yl)pyridine in compound **2** appeared to enhance its potency relative to the 1,10-phenanthroline moiety in compound **3** in all assays, with the exception of BChE inhibition, where compound **3** exhibited superior activity (Table 1).

### 2.2. 4-N-Phenylaminoquinoline Derivatives

**Figure 3:**
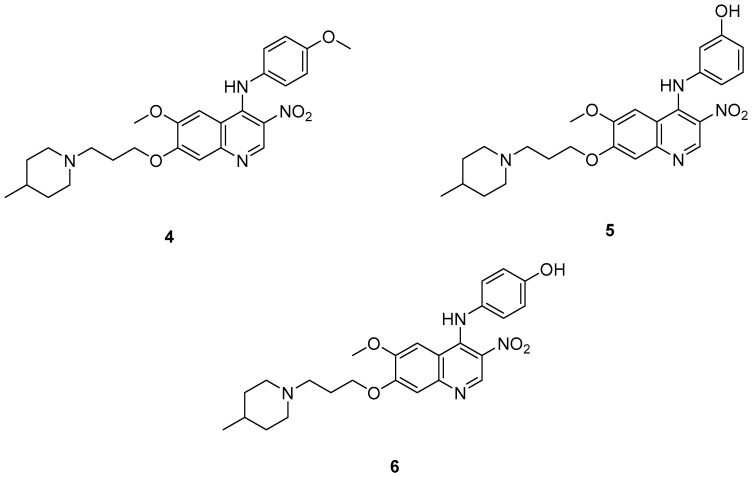
Chemical structures of 4-*N*-phenylaminoquinoline derivatives (**4**–**6**) [49].

Cai, R. et al. [49] developed and analyzed a series of new 4-*N*-phenylaminoquinoline derivatives. Their AChE inhibitory activities were assessed against electric eel acetylcholinesterase (eeAChE) and equine serum butyrylcholinesterase (eqBChE) using Ellman’s method, with galantamine as the reference compound. Compound **4**, featuring a para-methoxy group, exhibited the most potent inhibitory effect, with an IC_50_ value of 1.20 ± 0.18 μM, compared to galantamine (IC_50_ = 1.28 ± 0.01 μM). Moreover, compounds **5** and **6** showed remarkable results with IC_50_ values of 1.23 ± 0.03 and 1.40 ± 0.23 μM, respectively. In terms of inhibitory activity against eqBChE, compounds **4**, **5** and **6** (Figure 3) exhibited significant biological activities. Notably, compound **4** once again demonstrated the highest activity, with an IC_50_ value of 18.52 ± 1.21 μM. Compounds **5** and **6** also demonstrated considerable activity, with IC_50_ values of 22.11 ± 1.43 and 28.43 ± 4.70 μM, respectively. Galantamine was used as the reference compound (IC_50_ = 24.41 ± 2.01 μM). The selectivity index (SI) for AChE, calculated as IC_50_(eqBChE)/IC_50_(eeAChE), was calculated for compounds **4**, **5**, and **6** (15.43, 17.98, and 20.31, respectively), compared to galantamine (SI = 19.07). These values indicate that the inhibitory activity of these compounds is stronger toward AChE than BChE. Propidium iodide displacement (a selective PAS-AChE inhibitor) assay was conducted. From the results derived, compound **5** has been proved to be more efficient at displace propidium (25.80%) than donepezil (18.50%). DPPH radical scavenging activity was tested to assess their antioxidant effects. Compound **6** exhibited excellent DPPH radical scavenging activity (84% at 1 mg/mL) compared to the standard, ascorbic acid 97% (at 1 mg/mL). The results indicated that the radical scavenging activity of compound **6** increased with increasing concentration. Compound **6** exhibited a remarkable EC_50_ value of 0.328 μM, highlighting its strong antioxidant effect in the submicromolar range, comparable to that of ascorbic acid (EC_50_ = 0.095 μM). Compounds **4**, **5,** and **6** were selected for their chelating abilities toward Cu^2+^, Fe^2+^, Al^3+^, and Zn^2+^, using an UV spectrophotometer across a wavelength range of 200 nm to 600 nm. All synthesized compounds exhibited significant biometal chelation activities towards Al^3+^, Fe^2+^, Cu^2+^, and Zn^2+^ ions. Compound **6** was selected for molecular docking and shown to interact with the catalytic anionic site (CAS) and the peripheral anionic site (PAS) of both AChE and BChE. The presence of a 4-methoxy substitution appears to enhance activity against AChE more than BChE, as observed in compound **4** (IC_50_ = 1.20 ± 0.18 and 18.52 ± 1.21, respectively). Additionally, the presence of *para*-hydroxy substitution (compound **6**), appears to slightly reduce both AChE and BChE inhibitory activity. Although *para*-hydroxy substitution is considered crucial for DPPH scavenging activity, the other two compounds showed low interaction. Therefore, compound **6** stands out as the most effective, due to its ability to interact with the DPPH radical and inhibit both AChE and BChE (Table 2).

### 2.3. 1,2,3,4-Tetrahydroacridine Derivatives

**Figure 4:**
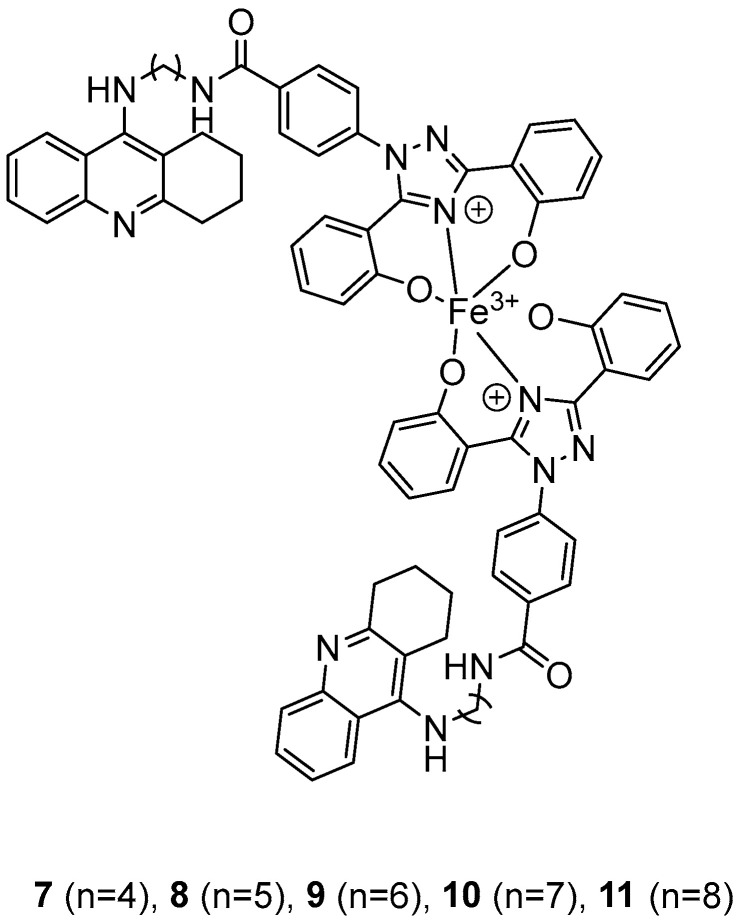
Chemical structures of 1,2,3,4-tetrahydroacridine derivatives (**7**–**11**) [50].

In their recent research, Wang, Y. et al. [50] synthesized novel 1,2,3,4-tetrahydroacridine derivatives and evaluated their potential for acetylcholinesterase from bovine serum (bAChE) inhibition, metal ion chelation, and antioxidant activity. All of the compounds exhibited excellent inhibitory effect against bAChE, with compounds **7** and **11** (Figure 4) showing lower IC_50_ values (13.5 ± 0.82 and 13.7 ± 0.21 μM, respectively) compared to the reference compound, tacrine (IC_50_ = 14.1 ± 0.33 μM). The results were determined by the ELISA method. To test their antioxidant activity, a DPPH assay was conducted. Compound **7** exhibited the highest antioxidant activity among the synthesized compounds, with 32.0 ± 0.23%. Compounds **8** and **9** also showed moderate activity, with 34.0 ± 0.56% and 39.0 ± 0.42%, respectively. However, none of the synthesized compounds demonstrated stronger antioxidant activity than the reference compound, deferasirox (23.0 ± 0.52%). To evaluate the cytotoxicity of the compounds, cell viability was assessed using the rat pheochromocytoma PC12 cell line through the 3-(4,5-dimethylthiazol-2-yl)-2,5-diphenyltetrazolium bromide (MTT) assay. Tacrine and deferasirox were included as reference compounds. Moreover, compound **11** showed low cytotoxicity and demonstrated effective protective activity against H_2_O_2_. An in vitro blood–brain barrier permeation assay was conducted by calculating cLogP values using the ChemDraw 17.0. The cLogP values of all compounds were greater than 5, suggesting their potential to cross the blood–brain barrier. They also tested the metal-chelating properties of compound **11** by using the UV–vis spectrometric method across a wavelength range of 200 to 500 nm. The results showed that compound **11** was able to bind Fe^3+^ and Cu^2+^ to form chelate metal complexes. Molecular modeling studies showed that compound **11** could simultaneously occupy the CAS and the PAS of the Torpedo californica variant of AChE (TcAChE). The chain length does not appear to significantly affect acetylcholinesterase (bAChE) inhibition, as both compound **7** (n = 4) and compound **11** (n = 11) exhibited the strongest activities. In addition, in the DPPH assay, an increase in chain length appears to enhance interaction with the radical, as observed in the results for compound **11**. As a result, compound **11** is considered the most potent, due to its strong acetylcholinesterase (bAChE) inhibitory activity and its ability to effectively scavenge DPPH radical (Table 3).

### 2.4. Deferiprone–Resveratrol Hybrids

**Figure 5:**
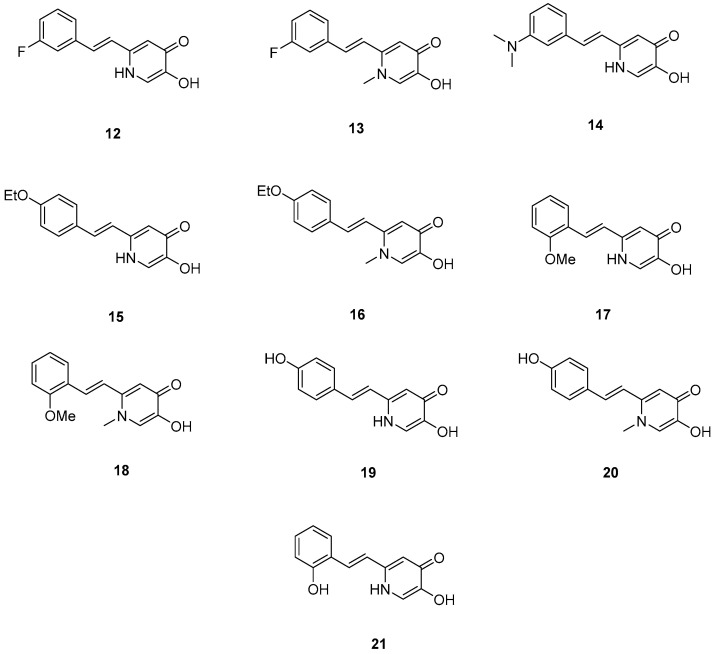
Chemical structures of 1,2,3,4-tetrahydroacridine derivatives (**12**–**21**) [51].

Xu, P. et al. [51], constructed a series of deferiprone–-resveratrol hybrids as multitarget-directed ligands (MTDLs). To assess the ligand affinity for the metal under biological conditions they used the pFe(III) value, which is defined as the negative logarithm of free iron concentration in solution. The pFe(III) values of all synthesized compounds were measured using a fluorescence-based method, with deferiprone being the reference compound (pFe(III) = 20.60). Most of the synthesized compounds exhibited excellent pFe(III) values, with compound **18** (Figure 5) showing the highest, at 20.58 ± 0.18. A Thioflavin-T (ThT) based fluorometric assay was used to test the ability of compounds to inhibit Aβ_1–42_ self-induced aggregation. Resveratrol and curcumin were used as reference compounds with IC_50_ values of 11.89 ± 2.52, and 18.73 ± 0.32 μM, respectively. Compounds **12**, **16**, **19,** and **20** demonstrated potent inhibitory activities with IC50 values of 18.97 ± 2.22, 8.94 ± 0.84, 10.72 ± 0.5, and 15.38 ± 0.30 μM, respectively. The antioxidant capacity of the compounds was assessed using the ABTS [2,2′-azino-bis(3-ethylbenzothiazoline-6-sulfonic acid)] radical scavenging assay, with Trolox (IC_50_ = 3.89 ± 0.09 μM) used as a reference compound. Compounds showed moderate to good radical scavenging activities, with compound **13** being the most potent with IC_50_ value of 1.31 ± 0.07 μM. Molecular docking studies were conducted and confirmed that compounds **16** and **19** efficiently interact with the C-terminus of Aβ_1–42_ by hydrogen bonds. It can be concluded that adding a methyl group at position-1 of the pyridine ring may improve the ability of some compounds, such as compound **16** compared to **15**, and compound **18** compared to **17**, to inhibit Aβ_1–42_ self-induced aggregation. However, this effect is not observed in compound **13** compared to **12**, or in compound **20** compared to **19**. The *para*-hydroxy substitution in compound **20** increases the inhibition of Aβ_1–42_ self-induced aggregation compared to the sterically hindered *ortho*-substitution in compound **21**. Additionally, the *ortho*-methoxy substitution (compound **17**) appears to enhance the inhibition of Aβ_1–42_ self-induced aggregation compared to the *ortho*-hydroxy substitution (compound **21**). The *para*-hydroxy substitution in compound **19**, a stronger electron-donating group compared to the *para*-ethoxy group in compound **15**, is associated with a lower IC_50_ value in the inhibition of Aβ_1–42_ self-induced aggregation. In the antioxidant assay, the presence of a methyl group at the 1-position of the pyridine ring was correlated with excellent radical scavenging activity. As expected, the compounds bearing two hydroxyl groups on the benzene ring (compounds **19**, **20**, and **21**) demonstrated greater antioxidant activities than Trolox. Among them, compound **20** showed the highest activity, which may be attributed to the non-sterically hindered *para*-substitution (Table 4).

### 2.5. Tacrine–(Hydroxybenzoyl-Pyridone) Hybrids

**Figure 6:**
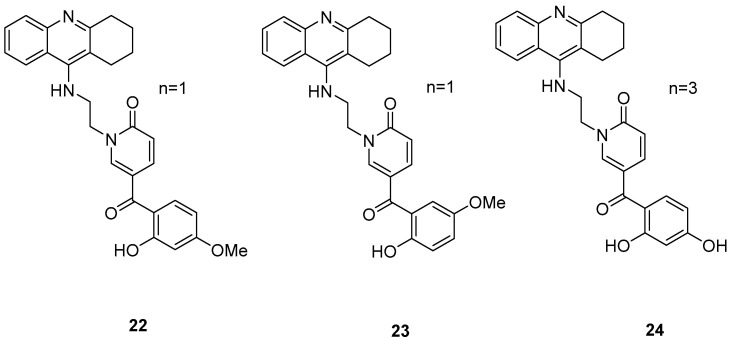
Chemical structures of tacrine-(hydroxybenzoyl-pyridone) hybrids (**22**–**24**) [52].

Three novel potentially site-activated multitarget tacrine–-(hydroxybenzoyl-pyridone) (TAC-HBP) hybrids were synthesized and assessed by Chand, K. et al. [52], as AChE inhibitors, antioxidants, and biometal chelators. The AChE inhibitory activity was evaluated by adaptation of the spectroscopic method described by Ellman. All the synthesized compounds exhibited excellent inhibitory effects, with compound **24** (Figure 6) being the most potent, with IC_50_ = 0.57 ± 0.05 μM. Tacrine was used as the reference compound, with an IC_50_ value of 0.31 ± 0.02 μM. In DPPH free radical scavenging (EC_50_), all the derivatives showed similar results, which significantly improved compared to the parent drug tacrine (EC_50_ > 1000 μM). Notably, compound **24** exhibited the strongest DPPH radical scavenging activity, which may be attributed to the 2′,4′-dihydroxy substitution on the aromatic ring (Table 5). In the AChE inhibitory assay, the results suggested that the length of the linker between the two coupled moieties may play a role in enhancing inhibitory potency against AChE. Compound **24**, which contains a four-methylene-unit linker, was the most potent and appears to fit well between the CAS and the PAS of the enzyme. As for the chelating capability against redox-active and/or Aβ-binding metal ions (Fe(III), Cu(II)), and Zn(II)) it has been proved that the HBP moiety is a moderate/good chelator of these biometals and is capable of forming complexes with β-phenol-keto coordination mode.

### 2.6. Diallyl Disulfide (DADS) Derivatives

**Figure 7:**
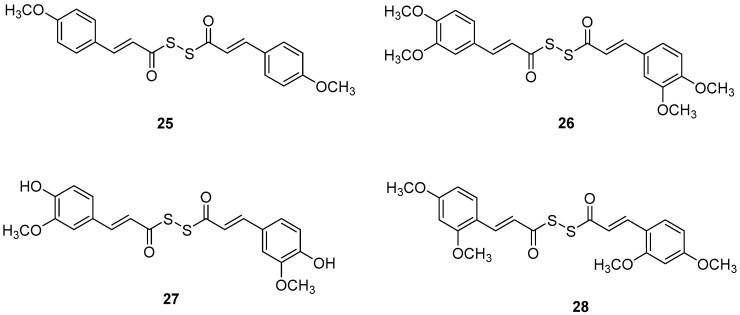
Chemical structures of tacrine-(hydroxybenzoyl-pyridone) hybrids (**25**–**28**) [53].

Manral, A. et al. [53], designed, synthesized, and evaluated a series of novel diallyl disulfide (DADS) derivatives. They tested the inhibition of self-mediated Aβ_1–42_ aggregation using *in vitro* assays with ThT to monitor and quantify the aggregation of Aβ_1–42_ synthetic peptide into fibrils. Curcumin was used as the reference compound (51.5 ± 2.68%). All the synthesized compounds showed high % inhibition, with compounds **26** and **27** (74.16 ± 2.10 and 71.41 ± 3.04, respectively) exhibiting the best effects. To investigate the inhibitory effect of the synthesized AChE and BuChE, they used modified Ellman’s method. Donepezil was the reference compound with an IC_50_ value of 8.71 ± 1.36 µM for BuChE assay and 0.049 ± 0.05 µM for AChE assay. In the AChE assay, the compounds exhibited moderate inhibitory activities, with compounds **26** and **27** (Figure 7) showing the most potent effects, having IC_50_ values of 13.64 ± 0.27 µM and 12.87 ± 0.81 µM, respectively. In the BuChE assay, compounds **26** and **27** once again demonstrated the most potent activities, with IC_50_ values of 0.056 ± 0.05 µM and 0.121 ± 0.06 µM, respectively (Table 6). The results suggest that substitutions on the aromatic ring significantly influence the inhibitory effects of the compounds on both AChE and BuChE. Compounds **26** and **27** were the most potent towards cholinesterase enzymes. Particularly, the activity ranging of the substituted groups on the ring is as follows: 3,4-dimethoxy > 4-hydroxy, 3-methoxy > 4-methoxy > 4-hydroxy. Consequently, the electron density of the aromatic ring appears to play a crucial role in the inhibitory activity of the compounds against cholinesterase enzymes. Moreover, the antioxidant activities of the synthesized compounds were evaluated through the oxygen radical absorbance capacity assay using fluorescein (ORAC-FL). The vitamin E analogue Trolox was used as a standard. All the synthesized compounds demonstrated excellent antioxidant activities, with compounds **26** and **27** showing the most potent results, exhibiting ORAC-FL values of 5.24 ± 0.37 µM and 5.86 ± 0.98 µM, respectively. Compounds **26** and **27** have a free -OH and -OCH_3_ group on the phenyl ring, which might be crucial to the radical scavenging ability. Compounds **26** and **27** were able to disaggregate Aβ fibrils formed by Cu^2+^-induced aggregation, achieving disaggregation rates of 80.9% and 78.5%, respectively, as further confirmed by transmission electron microscopy (TEM) analysis. Furthermore, the ability of compound **26** to chelate biometals like Cu^2+^ and Fe^2+^ by UV- visible spectrometry was studied. The results showed that compound **26** possesses a chelating ability and it is attributed to the presence of the dimethoxy group on the phenyl ring and keto-moieties in the core structure of the compound. Compound **26** can simultaneously bind to both bivalent cations. The stoichiometry of the compound **26**–Cu^2+^ complex was determined by the molar ratio method. The results indicated 1:1 stoichiometry for the complex. Molecular modeling studies showed that compounds **26** and **27** could simultaneously occupy the CAS and the PAS of AChE. Moreover, cytotoxicity assays performed in compounds **26** and **27** did not indicate toxicity and revealed that they have druglike properties.

### 2.7. 3-Schiff Base-4-Hydroxycoumarin Derivatives

**Figure 8:**
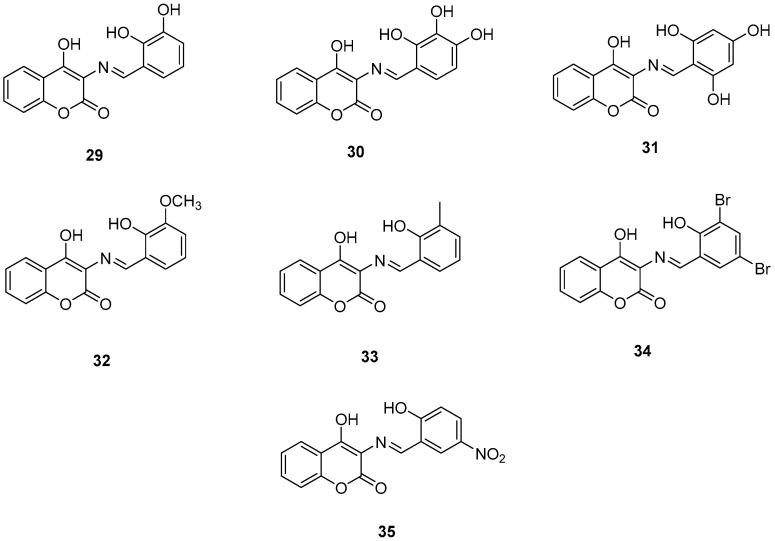
Chemical structures of 3-Schiff base-4-hydroxycoumarin derivatives (**29**–**35**) [54].

Wang, Z.-M. et al. [54] designed and synthesized a series of 3-Schiff base-4-hydroxycoumarin derivatives which were further evaluated for their monoamine oxidase inhibitory activities. All the synthesized compounds exhibited good inhibitory activities against hMAO-A and hMAO-B. Compound **30** showed the best hMAO-A inhibition, with IC_50_ value of 0.673 ± 0.011 µM. Iproniazid was used as the reference compound, with IC_50_ = 7.14 ± 0.38 µM. In the hMAO-B inhibitory assay, compound 4 exhibited the most potent inhibitory activity, with an IC_50_ value of 0.711 ± 0.013 µM, while the other compounds showed moderate to good activity. Pargyline and Iproniazid were the reference compounds, with IC_50_ values of 0.214 ± 0.036 µM and 8.54 ± 0.64 µM, respectively. The ability to inhibit self-induced Aβ_1–42_ aggregation was tested by using a ThT based fluorometric assay. All the compounds exhibited moderate to good results with compound **31** (Figure 8) exhibiting the highest efficacy (82.3 ± 6.6%). Curcumin and resveratrol were used as reference compounds, with 50.2 ± 5.9 and 67.3 ± 3.4%. Compounds **29**, **30**, **31,** and **35** were selected to investigate their ability to inhibit Cu^2+^-induced Aβ_1–42_ aggregation by the ThT-binding assay. The results indicate that these compounds could inhibit Cu^2+^-induced Aβ_1–42_ aggregation effectively. Additionally, they have been tested for their antioxidant activity through the ABTS and DPPH radical scavenging protocols. Trolox was the reference compound, and curcumin was determined for comparison. The best activity was observed for **35** with 1.57 trolox equivalents. For the DPPH radical scavenging assay resveratrol and curcumin were used as reference compounds with IC_50_ values of 137 ± 4 and 39.6 ± 2.1 µM, respectively. Among the tested derivatives, compounds **30** and **35** were the most potent with IC_50_ values of 45.8 ± 1.2 and 38.6 ± 2.0 µM, respectively (Table 7). Molecular docking and kinetic studies revealed that compound **30** was a reversible and noncompetitive MAO-B inhibitor. Moreover, these compounds showed low PC12 cells toxicity. To determine the ability of the compounds to cross the BBB, a parallel artificial membrane permeation assay for the BBB (PAMPA-BBB) was used. It can be concluded that compound **35** has shown higher P_e_ values than 4.5 being capable of crossing the blood–brain barrier, but compound **30** was uncertain for BBB permeation (CNS±). To assess BBB permeability, the compounds were categorized as follows: those with Pe (10^−6^ cm/s) > 4.5 were classified as having high BBB permeability (CNS+); those with Pe < 2.1 as having low permeability (CNS−); and compounds with Pe values between 2.1 and 4.5 as having uncertain BBB permeability (CNS±). Compound **30** exhibited the most potent hMAO-A and hMAO-B inhibitory activity, likely due to the presence of three hydroxyl groups in the *ortho*, *meta*, and *para* positions. Methoxy and methyl substitutions, which are weaker electron-donating groups than hydroxy, at the *meta* position appear to reduce hMAO-A inhibitory activity. For their antioxidant activity compound **35** had the best results with an additional nitro group in 5-position of the benzaldehyde ring. *Ortho* or *para* substitution of hydroxyl groups on the benzene ring favors the ABTS radical scavenging activity.

### 2.8. Selegiline Derivatives

**Figure 9:**
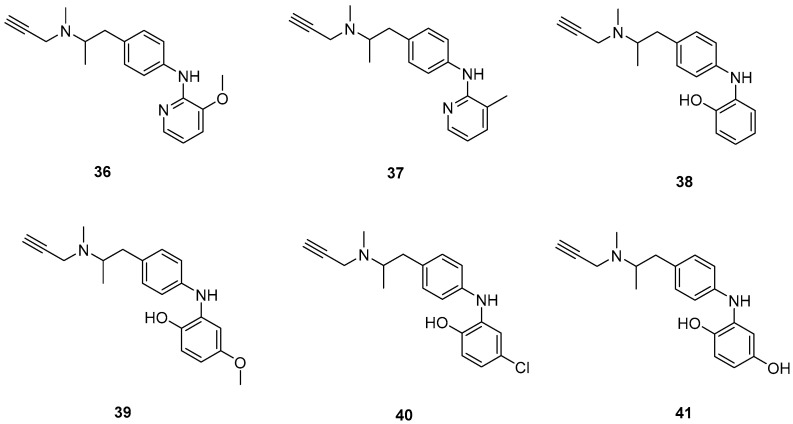
Chemical structures of selegiline derivatives (**36**–**41**) [55].

Xie, S. et al. [55] designed and synthesized a new series of selegiline derivatives. In vitro inhibition studies of hMAO-A and hMAO-B were conducted, using clorgyline and pargyline as reference compounds. All the synthesized compounds showed excellent hMAO-B inhibitory activity, with compound **38** (Figure 9) being the most potent (IC_50_ = 0.21 ± 0.04 μM), comparable to the reference compound pargyline (IC_50_ = 0.1880 μM). Compound **38** has no substituents other than the hydroxy group on the phenyl ring. It appears that substitution at the *ortho* and *meta* positions relative to the hydroxyl group on the benzene ring is not essential for the inhibitory activity. The presence of a strong electron-donating group in the *ortho* position, as in compound **36**, enhances hMAO-A and hMAO-B inhibitory activity compared to compound **37**, bearing a methyl group in the same position. The results indicate that the compounds also exhibit effective MAO-A inhibition, with compound **40** showing the most potent activity (IC_50_ = 0.70 ± 0.01 μM). The antioxidant activities using the oxygen radical absorbance capacity (ORAC-FL) assay was studied, with fluorescein as the probe and the vitamin E analogue Trolox as the standard. The results revealed that all the synthesized compounds possessed excellent antioxidant capacity with ORAC-FL values of 1.49–5.65 Trolox equivalents. In general, compound **41**, bearing two hydroxy groups on the phenyl ring, exhibited the best result (5.67 Trolox equivalents). The metal-chelating ability of compound **38** was investigated using UV–vis spectrometry. Based on the shifts in the absorption spectra, it was suggested that compound **38** can interact with iron(II) and zinc(II). High-resolution mass spectrometry was performed to determine the stoichiometry of the compound **38**–Cu(II) complex. The results showed that the stoichiometry of the complex is 2:1. Additionally, the biometal chelating ability of compound **38**, was evaluated and the results demonstrated that it can effectively inhibit Cu(II)-induced Aβ_1–42_ aggregation. To determine whether the compounds could cross the BBB, a PAMPA-BBB was used. In fact, compounds with a permeability above 4.7 × 10^−6^ cm/s are likely to cross the BBB via passive diffusion (CNS+), while those with Pe values between 1.8 × 10^−6^ cm/s and 4.7 × 10^−6^ cm/s were classified as having uncertain BBB permeability (CNS+/−). Compounds **36**, **37**, **38** and **39** can cross the BBB (Table 8).

### 2.9. Scutellarein Carbamate Derivatives

**Figure 10:**
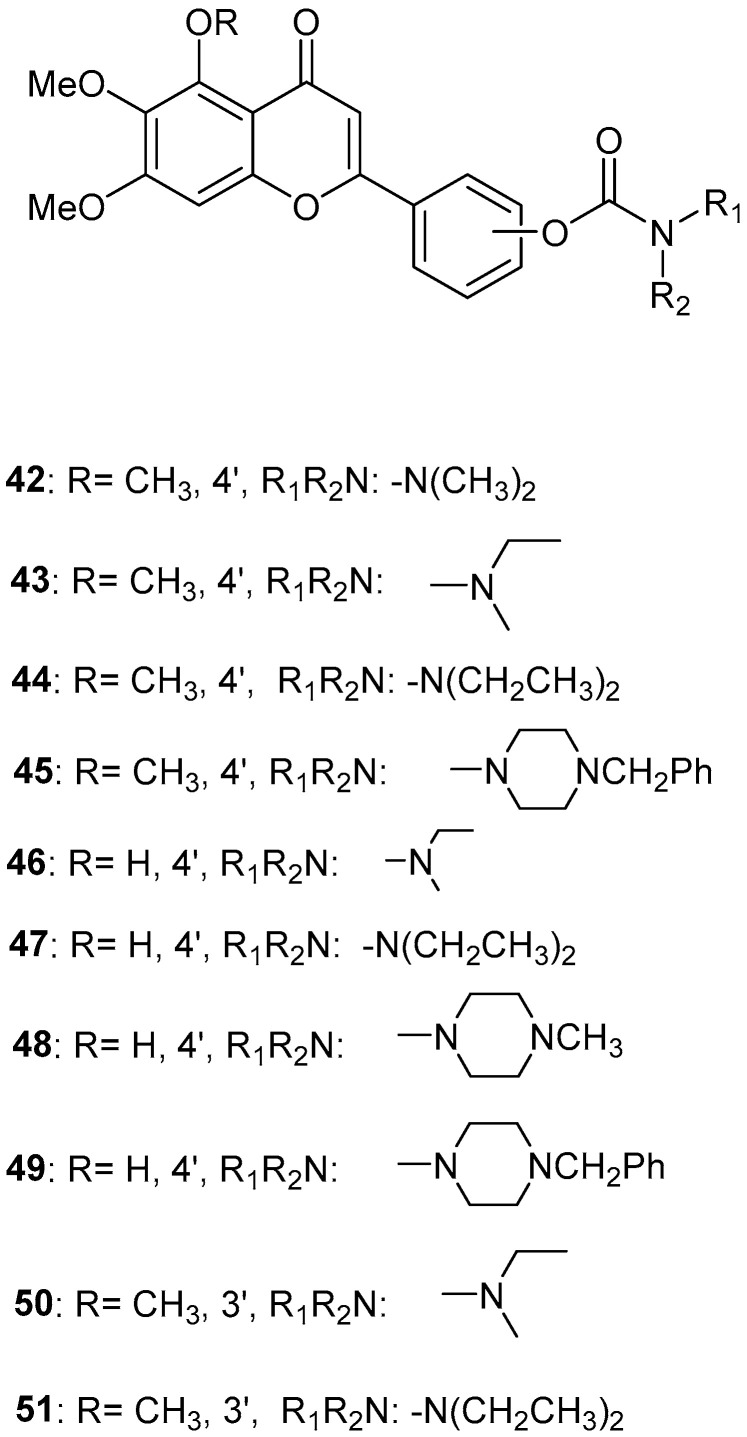
Chemical structures of scutellarein carbamate derivatives (**42**–**51**) [56].

Sang, Z. et al. [56] produced a series of scutellarein carbamate derivatives as multifunctional agents for the treatment of AD. To determine the AChEand BuChE inhibitory activities of the synthesized compounds the modified Ell man method was applied using AChE from rat cortex homogenate (RatAChE) and BuChE from rat serum (RatBuChE). Rivastigmine was used as a reference compound with IC_50_ = 5.6 ± 0.02 μM for AChE and 1.4 ± 0.01 μM for BuChE. All the synthesized compounds exhibited good inhibitory activity against AChE with compounds **44** and **47** (Figure 10) showing the best results with IC_50_ values of 0.34 ± 0.03 μM and 0.57 ± 0.02 μM, respectively. Both compounds contain the *N*,*N*-diethylcarbamate moiety in their structure. Almost all the compounds exhibited weak activity against BuChE and showed higher selectivity towards AChE over BuChE. Compound **43** had the best results against BuChE with IC_50_ = 6.2 ± 0.21 μM. Substitutions at the 5-position of the flavonoid core significantly influenced AChE inhibitory activity; derivatives with a 5-methoxy group (compounds **42**–**45** and **50**–**51**) exhibited greater activity than those with a 5-hydroxyl group (**46**–**49**). The results also showed that placing the carbamate group at the 4′-position (compounds **42**–**45** and **46**–**49**) led to stronger inhibitory activity compared to placement at the 3′-position (compounds **50**–**51**). This suggests that the carbamate group at the 4′-position is more favorably oriented to interact with the CAS of AChE. The ability of compounds **44** and **47** to chelate biometals such as Cu^2+^, Zn^2+^, Al^3+^ and Fe^2+^ was evaluated by UV–vis spectrometry. The chelating ability can be attributed to the 5-hydroxyl and 4-carbonyl groups at the flavonoid nucleus. The stoichiometry of the compound **47**–Cu^2+^ complex was calculated and the results showed 1:1 stoichiometry for the complex. In conclusion, compounds **44** and **47** were the most potent and were selected for further studies on their metal-chelating abilities. The kinetic characterization on compound **47** suggests a mixed-type inhibition, binding to both CAS and PAS of AChE, which was consistent with the result of the molecular modeling study. The compounds have been further evaluated for their antioxidant activity, by using the well-established ORAC-FL method (Oxygen Radicals Absorbance Capacity by Fluorescence). The vitamin E analogue Trolox was used as a standard, and the results were expressed as Trolox equivalents. Compounds **46**, **47**, **48** and **49** exhibited the highest antioxidant activities with ORAC-FL values of 1.1 ± 0.05, 1.3 ± 0.02, 1.0 ± 0.03 and 0.9 ± 0.01, respectively (Table 9). These compounds contain a hydroxyl group at position 5 of the flavonoid core and exhibited superior antioxidant activity compared to compounds **42**–**45**, which contain a 5-methoxyl group. The neuroprotective potential of the selected carbamate derivatives under oxidative stress was evaluated by assessing their ability to protect PC12 cells from hydrogen peroxide-induced injury. Cell viability was determined using the MTT assay. Compound **47** exhibited remarkable neuroprotective effects, and the cell viability was 78.5%. The possible in vivo BBB permeability of compound **47** was evaluated with the PAMPA–BBB. Compound **47** exhibited a permeability (Pe × 10^−6^ cm/s) of 8.42 ± 0.37, suggesting its potential to cross the BBB and reach targets within the CNS. The protective effects of compound **47** on scopolamine-induced cognitive impairment in mice, were evaluated using the Y-maze test. The results revealed that compound **47** can decrease the vitality of AChE and increase the vitality of ChAT in the hippocampus of mice.

### 2.10. Donepezil-Related Derivatives

**Figure 11:**
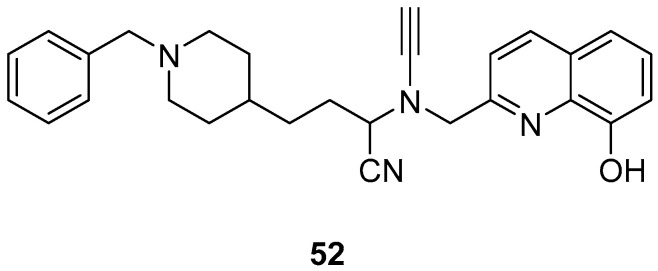
Chemical structures of donepezil-related derivative (**52**) [57].

Wu, M.-Y. et al. [57] constructed a series of donepezil-related derivatives possessing metal chelating properties, and being capable of targeting different enzymatic systems related to AD (cholinesterases, ChEs, and monoamine oxidase A, MAO-A). Compound **52** (Figure 11) showed the best activity, inhibiting AChE activity with an IC_50_ value of 0.029 μM. Additionally, compound **52** demonstrated excellent inhibitory activity (IC_50_ = 0.039 μM) against the BuChE being more potent than the reference compound, donepezil (IC_50_ = 7.5 μM). Compound **52** was found to be a selective MAO-A (vs MAO-B) inhibitor with IC_50_ = 10.1 μM. An in vitro assay to establish its metal-chelating properties of compound **52** was applied using UV–vis spectrometry. The results showed that compound **52** was able to interact with Cu^2+^, Fe^2+^ and Zn^2+^. The results indicated a 1:2 stoichiometric complexation between compound **52** and both Cu^2+^ and Zn^2+^, revealing a metal-to-compound **52** ratio of 1:2. The antioxidant activity was evaluated in vitro using the linoleic acid assay and 2,2′-azobis(2-amidinopropane) dihydrochloride (AAPH). The tested compounds exhibited 50–60% activity compared to the reference compound Trolox, which showed 100–120% activity. Finally, compound **52** exhibited weak radical scavenging activity (RSA) in the DPPH assay, compared to Trolox (Table 10).

## 3. Conclusions

Metal-chelating agents have gained significant importance in medicine in recent years, and many researchers are synthesizing them for the treatment of AD. Oxidative stress has emerged as an important factor involved in the pathogenesis of Alzheimer’s disease and consequently, antioxidants can be a key factor in AD treatment. In this review, the role of bifunctional metal chelators with antioxidant properties for the treatment of Alzheimer’s was described. It was found that several potent inhibitors exhibited good to excellent activity against AChE, BuChE, hMAO-A, and hMAO-B, as well as in antioxidant assays and self-induced Aβ_1–42_ aggregation. Several of the synthesized compounds exhibited excellent AChE inhibitory activity, with compounds **4**, **5**, **7**, **11**, **16**, **19**, and **43**–**48** showing the highest potency, as indicated by IC_50_ values (1.20 ± 0.18, 1.23 ± 0.03, 13.5 ± 0.82, 13.7 ± 0.21, 8.94 ± 0.84, 10.72 ± 0.5, 1.54 ± 0.04, 0.34 ± 0.03, 1.04 ± 0.05, 2.10 ± 0.05, 0.57 ± 0.02 and 5.0 ± 0.05 μM, respectively) lower than those of the reference compounds. Most of these compounds possess substituted hydroxyl and methoxy aromatic rings. Several compounds also demonstrated good BuChE inhibitory activity; however, the results indicated greater selectivity toward AChE. Compounds **29**, **30**, **34**, **35**, and **39**–**41** exhibited excellent dual inhibitory activity against hMAO-A and hMAO-B. Compounds **29**, **30**, **34**, and **35** possess at least one hydroxyl group on the aromatic ring, with compound **30** exhibiting the most potent activity, likely due to the presence of three hydroxyl groups located at the *ortho*, *meta*, and *para* positions. This may be attributed to the stronger electron-donating ability of the hydroxyl group compared to other substituents. Compound **35**, having a nitro moiety on the aromatic ring, presented the best antioxidant activity. In the self-induced Aβ_1–42_ aggregation assay, compounds **16**, **19**, **20**, **25**–**28**, **31**, and **35** showed the most effective inhibition compared to the reference compounds. Substitution with methoxy or hydroxyl groups on the phenyl ring of compounds **25**–**28** seems to contribute to the activity. In general, hydroxyl groups on aromatic rings enhance the activity of the synthesized compounds. Additionally, compounds with good antioxidant activities possess free hydroxyl, methoxy or ethoxy moieties. Compounds **1**–**3**, **6**, **10**, **11**, **13**, **14**, **19**–**25**, **29**, **30,** and **35**–**52** exhibit potent dual metal- chelating, and antioxidant properties for the treatment of AD. Regarding metal-chelating properties, most of the compounds can interact with Fe^2+^ and Cu^2+^ ions, while a few also show affinity for Zn^2+^ and Al^3+^. A considerable number of the synthesized compounds exhibited good antioxidant activity, and their biometal chelating abilities also yielded promising results. All the above discussed results have been included in Table 11, summarizing the metal-binding or metal-related properties of the discussed compounds (Table 11).

## Figures and Tables

**Table 1:** Bioassay results for compounds **1**–**3** and reference compounds [48].

Compound	AChE Assay (IC_50_ μΜ)	BChE Assay (IC_50_ μΜ)	DPPH Assay (IC_50_ μΜ)	ABTS Assay (IC_50_ μΜ)
**1**	59.52 µM	55.56 µM	0.927 µM	1.15 µM
**2**	33.07 µM	396.83 µM	0.245 µM	0.070 µM
**3**	141.02 µM	0.056 µM	0.401 µM	0.721 µM
Reference: Galantamine	1.67 µM	2.13 µM	-	-
Reference: Ascorbic acid	-	-	3.62 µM	2.03 µM

**Table 2:** Bioassay results for compounds **4**–**6** and reference compounds [49].

Compound	eeAChE IC_50_ (μM)	eqBChE IC_50_ (μM)	DPPH Assay % (at 1 mg/mL)	Propidium Iodide Displacement (%)	SI
**4**	1.20 ± 0.18 µM	18.52 ± 1.21 µM	5	14.85 ± 0.59	15.43
**5**	1.23 ± 0.03 µM	22.11 ± 1.43 µM	-	25.80 ± 1.37	17.98
**6**	1.40 ± 0.23 µM	28.43 ± 4.70 µM	84	13.78 ± 0.96	20.31
Reference: Donepezil	-	-	-	18.50 ± 1.13	-
Reference: Galantamine	1.28 ± 0.01 µM	24.41 ± 2.01 µM	-	-	19.07
Reference: Ascorbic acid	-	-	97	-	-

**Table 3:** Bioassay results for compounds **7**–**11** and reference compounds [50].

Compound	bAChEb IC_50_ (μM)	DPPH Assay %
**7**	13.5 ± 0.82 µM	32 ± 0.23
**8**	17.8 ± 0.21 µM	34 ± 0.56
**9**	15.4 ± 0.27 µM	39 ± 0.42
**10**	16.1 ± 0.36 µM	49 ± 0.31
**11**	13.7 ± 0.21 µM	56 ± 0.69
Reference: Tacrine	14.1 ± 0.33 µM	-
Reference: Deferasirox	-	23 ± 0.52

**Table 4:** Bioassay results for compounds **12**–**21** and reference compounds [51].

Compound	Aβ_1–42_ IC_50_ (μM)	ABTS Assay IC_50_ (μM)	pFe(III)
**12**	18.97 ± 2.22	6.29 ± 0.57	20.24 ± 0.05
**13**	19.44 ± 0.31	1.31 ± 0.07	20.12 ± 0.40
**14**	29.22 ± 3.60	3.41 ± 1.15	20.06 ± 0.13
**1** **5**	23.39 ± 4.64	8.12 ± 0.49	20.12 ± 0.26
**1** **6**	8.94 ± 0.84	4.02 ± 0.34	19.00 ± 0.16
**17**	16.50 ± 1.16	6.74 ± 0.57	20.22 ± 0.16
**18**	12.48 ± 0.95	4.17 ± 0.51	20.58 ± 0.18
**19**	10.72 ± 0.5	1.73 ± 0.14	19.60 ± 0.29
**20**	15.38 ± 0.30	1.37 ± 0.01	20.05 ± 0.095
**21**	21.76 ± 1.58	2.21 ± 0.15	20.17 ± 0.16
Reference: Resveratrol	11.89 ± 2.52	0.76 ± 0.02	-
Reference: Curcumin	18.73 ± 0.32	-	-
Reference: Deferiprone	-	-	20.60
Reference: Trolox	-	3.89 ± 0.09	-

**Table 5:** Bioassay results for compounds **22**–**24** and reference compound [52].

Compound	EeAChE (IC_50_, μM)	DPPH Scavenging (EC_50_, μM)
**22**	0.78 ± 0.01 µM	249 ± 2 µM
**23**	0.71 ± 0.03 µM	213 ± 2 µM
**24**	0.57 ± 0.05 µM	204 ± 2 µM
Reference: Tacrine	0.31 ± 0.02 µM	>1000 µM

**Table 6:** Bioassay results for compounds **25**–**28** and reference compounds [53].

Compound	Inhibition of Self-Mediated Aβ_1–42_ Aggregation (%)	DPPH Scavenging (EC_50_, μM)	AChE (µM)	BuChE (µM)	ORAC (µM of Trolox Equivalents)
**25**	63.54 ± 4.79%	249 ± 2 µM	0.22 ± 0.06 µM	15.98 ± 0.34 µM	4.29 ± 0.65 µM
**26**	74.16 ± 2.10%	213 ± 2 µM	0.056 ± 0.05 µM	13.64 ± 0.27 µM	5.24 ± 0.37 µM
**27**	71.41 ± 3.04%	204 ± 2 µM	0.121 ± 0.06 µM	12.87 ± 0.81 µM	5.86 ± 0.98 µM
**28**	68.97 ± 3.45%	>1000 µM	0.142 ± 0.03 µM	14.89 ± 0.98 µM	5.09 ± 0.72 µM
Reference: Curcumin	51.5 ± 2.68%	-	-	-	-
Reference: Donepezil	-	-	0.049 ± 0.05 µM	8.71 ± 1.36 µM	-

**Table 7:** Bioassay results for compounds **29**–**35** and reference compounds.

Compound	hMAO-A (μM)	hMAO-B (μM)	Self-Induced Aβ_1–42_ Aggregation Inhibition	ABTS	DPPH Scavenging Activities IC_50_ (μM)	Permeability (P_e_ × 10^−6^ cm s^−1^)
**29**	4.42 ± 0.43	2.92 ± 0.11	66.0 ± 2.7	1.27	48.5 ± 2.7	-
**30**	0.673 ± 0.011	0.711 ± 0.013	60.1 ± 4.0	1.34	45.8 ± 1.2	4.16 ± 0.33
**31**	4.68 ± 0.66	17.4 ± 1.2	82.3 ± 6.6	1.36	55.3 ± 6.6	-
**32**	6.81 ± 0.36	11.2 ± 0.8	47.7 ± 5.5	0.82	84.3 ± 5.5	-
**33**	6.24 ± 0.67	3.18 ± 0.26	57.3 ± 1.1	1.19	78.5 ± 2.1	-
**34**	4.97 ± 0.41	0.851 ± 0.047	57.3 ± 2.1	0.30	-	-
**35**	3.78 ± 0.62	1.32 ± 0.18	73.5 ± 6.1	1.57	38.6 ± 2.0	9.95 ± 0.21
Reference: Pargyline	-	0.214 ± 0.036	-	-	-	-
Reference: Iproniazid	7.14 ± 0.38	8.54 ± 0.64	-	-	-	-
Reference: Resveratrol	-	-	67.3 ± 3.4	-	137 ± 4	-
Reference: Curcumin	-	-	50.2 ± 5.9	1.46	39.6 ± 2.1	-

**Table 8:** Bioassay results for compounds **36**–**42** and reference compounds.

Compound	hMAO-A (μM)	hMAO-B (μM)	ORAC	P_e_ (×10^−6^ cm s^−1^)
**36**	3.41 ± 0.02	0.31 ± 0.03	1.49	12.8 ± 0.7
**37**	5.53 ± 0.04	0.52 ± 0.05	1.78	12.1 ± 0.8
**38**	5.37 ± 0.03	0.21 ± 0.04	4.20	11.5 ± 0.5
**39**	1.02 ± 0.05	0.31 ± 0.02	3.81	11.7 ± 0.5
**40**	0.70 ± 0.01	0.31 ± 0.06	4.44	-
**41**	1.03 ± 0.06	0.43 ± 0.02	5.67	3.1 ± 0.3
Reference: Clorgyline	0.0041	1.32 ± 0.18	-	-
Reference: Pargyline	-	0.1880	-	-
Reference: Selegiline	70.2 ± 3.8	0.0185 ± 0.002	-	-

**Table 9:** Bioassay results for compounds **42**–**51** and reference compounds.

Compound	AChE (μM)	BuChE (μM)	ORAC	P_e_ (×10^−6^ cm s^−1^)
**42**	8.24 ± 0.32	7.3 ± 0.11	0.32 ± 0.01	-
**43**	1.54 ± 0.04	6.2 ± 0.21	0.34 ± 0.02	-
**44**	0.34 ± 0.03	8.2 ± 0.72	0.36 ± 0.01	-
**45**	1.04 ± 0.05	37.2 ± 0.23	0.32 ± 0.02	-
**46**	2.10 ± 0.05	38.1 ± 0.25	1.1 ± 0.05	-
**47**	0.57 ± 0.02	22.6 ± 0.22	1.3 ± 0.02	8.42 ± 0.37
**48**	5.0 ± 0.05	>100	1.0 ± 0.03	-
**49**	9.25 ± 0.06	>100	0.9 ± 0.01	-
**50**	7.37 ± 0.11	38.1 ± 0.12	0.32 ± 0.03	-
**51**	6.11 ± 0.07	30.1 ± 0.22	0.31 ± 0.01	-
Reference: Rivastigmine	5.6 ± 0.02	1.4 ± 0.01	-	-

**Table 10:** Bioassay results for compound **52** and reference compounds.

Compound	hrAChE IC_50_ (μM)	hrBuChE IC_50_ (μM)	hrMAO-A (μM)	AAPH (%)
**52**	0.029 ± 0.003	0.039 ± 0.003	10.1 ± 1.1	50–60
Reference: Donepezil	0.009 ± 0.001	7.5 ± 0.8	>1000	-
Reference: Trolox	-	-	-	100–120

**Table 11:** Summary of metal-binding and metal-related properties of the discussed compounds.

Compound	Metal-Related Properties
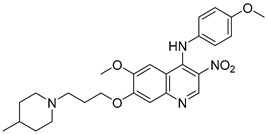 (**4**)	Chelating abilities toward Cu^2+^, Fe^2+^, Al^3+^ and Zn^2+^ using a UV spectrophotometer with wavelength ranging from 200 nm to 600 nm
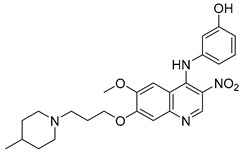 (**5**)	Chelating abilities toward Cu^2+^, Fe^2+^, Al^3+^ and Zn^2+^ using a UV spectrophotometer with wavelength ranging from 200 nm to 600 nm
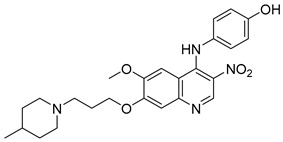 (**6**)	Chelating abilities toward Cu^2+^, Fe^2+^, Al^3+^ and Zn^2+^ using a UV spectrophotometer with wavelengths ranging from 200 nm to 600 nm
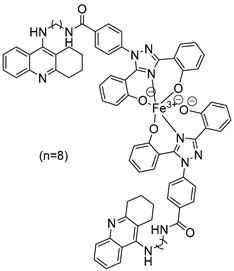 (**11**)	Chelating abilities toward Fe^3+^ and Cu^2+^ using a UV–vis spectrometric method with wavelength ranging from 200 to 500 nm.
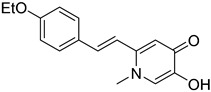 (**16**)	pFe(III) values using a fluorescence-based method
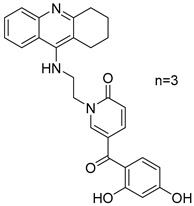 (**24**)	Chelating abilities toward Fe^2+^, Cu^2+^ and Zn^2+^ by spectrophotometric absorption with wavelengths ranging from 250 to 500 nm.
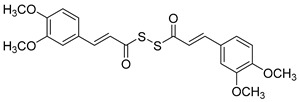 (**26**)	Chelating abilities toward Cu^2+^ and Fe^2+^ by UV- visible spectrometry with wavelengths ranging from 200 to 500 nm.
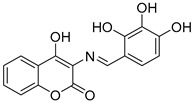 (**30**)	Chelating abilities toward Cu^2+^ by UV-vis spectrometry with wavelengths ranging from 200 to 500 nm.
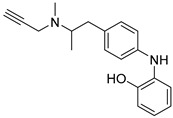 (**38**)	Chelating abilities toward Fe^2+^ and Zn^2+^ by UV–vis spectrometry with wavelengths ranging from 200 to 600 nm.
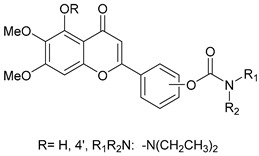 (**47**)	Chelating abilities toward Cu^2+^, Zn^2+^, Al^3+^ and Fe^2+^ by UV-vis spectrometry with wavelengths ranging from 200 to 480 nm.
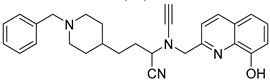 (**52**)	Chelating abilities toward Cu^2+^, Fe^2+^ and Zn^2+^ by UV-vis spectrometry with wavelengths ranging from 220 to 300 nm.

## Abbreviations

The following abbreviations are used in this manuscript:

Aβ	Amyloid-β
AAPH	2,2′-Azobis(2-Amidinopropane) Dihydrochloride
AChE	Acetylcholinesterase
AD	Alzheimer Dease
ADNC	Alzheimer Dease Neuropathological Change
APP	Amyloid Precursor Protein
BBB	Blood Brain Barrier
BChE	Butyrylcholinesterase
CAS	Catalytic Anionic Site
ChAT	Choline Acetyltransferase
CNS	Central Nervous System
DADS	Diallyl Disulfide Derivatives
DPPH	2,2-Diphenyl-1-Picrylhydrazyl
eeAChE	Electric Eel Acetylcholinesterase
EOAD	Early-Onset Alzheimer’s Disease
eqBChE	Equine Serum Butyrylcholinesterase
F-actin	Fibrillar Actin
G-actin	Globular Actin
HBP	Hydroxybenzoyl-Pyridone
hMAO-A	Human Monoamine Oxidase A
hMAO-B	Human Monoamine Oxidase B
LOAD	Late-Onset Alzheimer Dease
MTDLs	Multitarget-Directed Ligands
NFT	Neurofibrillary Tangle
ORAC-FL	Oxygen Radical Absorbance Capacity by Fluorescein
PAMPA	Parallel Artificial Membrane Permeation Assay
PART	Primary Age-related Tauopathy
PAS	Peripheral Anionic Site
PHFs	Paired Helical Filaments
RSA	Radical Scavenging Activity
RNS	Reactive Nitrogen Species
ROS	Reactive Oxygen Species
SI	Selectivity Index
TAC-HBP	Tacrine-(Hydroxybenzoyl-Pyridone)
TcAChE	Torpedo Californica Variant of AChE
TEM	Transmission Electron Microscopy
ThT	Thioflavin-T
VILIP-1	Visinin-Like Protein-1

## References

[1] Huang L.-K., Chao S.-P., Hu C.-J. (2020). Clinical trials of new drugs for Alzheimer disease. J. Biomed. Sci..

[2] Long J.M., Holtzman D.M. (2019). Alzheimer Disease: An Update on Pathobiology and Treatment Strategies. Cell.

[3] Scheltens P., De Strooper B., Kivipelto M., Holstege H., Chételat G., Teunissen C.E., Cummings J., van der Flier W.M. (2021). Alzheimer’s disease. Lancet.

[4] Mielke M.M. (2018). Sex and Gender Differences in Alzheimer’s Disease Dementia. Psychiatr. Times.

[5] Gallardo G., Holtzman D.M. (2019). Amyloid-β and Tau at the Crossroads of Alzheimer’s Disease. Adv. Exp. Med. Biol..

[6] Tarasoff-Conway J.M., Carare R.O., Osorio R.S., Glodzik L., Butler T., Fieremans E., Axel L., Rusinek H., Nicholson C., Zlokovic B.V. (2015). Clearance systems in the brain—Implications for Alzheimer disease. Nat. Rev. Neurol..

[7] Andrade-Guerrero J., Santiago-Balmaseda A., Jeronimo-Aguilar P., Vargas-Rodríguez I., Cadena-Suárez A.R., Sánchez-Garibay C., Pozo-Molina G., Méndez-Catalá C.F., Cardenas-Aguayo M.-D., Diaz-Cintra S. (2023). Alzheimer’s Disease: An Updated Overview of Its Genetics. Int. J. Mol. Sci..

[8] Van Cauwenberghe C., Van Broeckhoven C., Sleegers K. (2016). The genetic landscape of Alzheimer disease: Clinical implications and perspectives. Genet. Med..

[9] Xu L., Liu R., Qin Y., Wang T. (2023). Brain metabolism in Alzheimer’s disease: Biological mechanisms of exercise. Transl. Neurodegener..

[10] Yiannopoulou K.G., Papageorgiou S.G. (2020). Current and Future Treatments in Alzheimer Disease: An Update. J. Central Nerv. Syst. Dis..

[11] Wu D., Chen Q., Chen X., Han F., Chen Z., Wang Y. (2023). The blood–brain barrier: Structure, regulation and drug delivery. Signal Transduct. Target. Ther..

[12] Sousa J.A., Bernardes C., Bernardo-Castro S., Lino M., Albino I., Ferreira L., Brás J., Guerreiro R., Tábuas-Pereira M., Baldeiras I. (2023). Reconsidering the role of blood-brain barrier in Alzheimer’s disease: From delivery to target. Front. Aging Neurosci..

[13] Sadigh-Eteghad S., Sabermarouf B., Majdi A., Talebi M., Farhoudi M., Mahmoudi J. (2015). Amyloid-Beta: A Crucial Factor in Alzheimer’s Disease. Med Princ. Practice.

[14] Chen J., Chen J.-S., Li S., Zhang F., Deng J., Zeng L.-H., Tan J. (2023). Amyloid Precursor Protein: A Regulatory Hub in Alzheimer’s Disease. Aging Dis..

[15] Sehar U., Rawat P., Reddy A.P., Kopel J., Reddy P.H. (2022). Amyloid Beta in Aging and Alzheimer’s Disease. Int. J. Mol. Sci..

[16] Czuczwar S.J., Kocki J., Miziak B., Bogucki J., Bogucka-Kocka A., Pluta R. (2024). Alpha-, Beta-, and Gamma-Secretase, Amyloid Precursor Protein, and Tau Protein Genes in the Hippocampal CA3 Subfield in an Ischemic Model of Alzheimer’s Disease with Survival up to 2 Years. J. Alzheimer’s Dis..

[17] Kumar D., Anand P., Singh S. (2025). Proposed Therapeutic Strategy to Combat Alzheimer’s Disease by Targeting Beta and Gamma Secretases. Curr. Alzheimer Res..

[18] Blennow K., de Leon M.J., Zetterberg H. (2006). Alzheimer’s disease. Lancet.

[19] Hampel H., Hardy J., Blennow K., Chen C., Perry G., Kim S.H., Villemagne V.L., Aisen P., Vendruscolo M., Iwatsubo T. (2021). The Amyloid-β Pathway in Alzheimer’s Disease. Mol. Psychiatry.

[20] Busche M.A., Hyman B.T. (2020). Synergy between amyloid-β and tau in Alzheimer’s disease. Nat. Neurosci..

[21] Zheng H., Sun H., Cai Q., Tai H.-C. (2024). The Enigma of Tau Protein Aggregation: Mechanistic Insights and Future Challenges. Int. J. Mol. Sci..

[22] Carrillo-Mora P., Luna R., Colín-Barenque L. (2014). Amyloid Beta: Multiple Mechanisms of Toxicity and Only Some Protective Effects?. Oxidative Med. Cell. Longev..

[23] Yu Y., Yu S., Battaglia G., Tian X. (2024). Amyloid—β in Alzheimer’s disease: Structure, toxicity, distribution, treatment, and prospects. Ibrain.

[24] Metaxas A., Kempf S. (2016). Neurofibrillary tangles in Alzheimer′s disease: Elucidation of the molecular mechanism by immunohistochemistry and tau protein phospho-proteomics. Neural Regen. Res..

[25] Brion J.-P. (1998). Neurofibrillary Tangles and Alzheimer’s Disease. Eur. Neurol..

[26] De Ture M.A., Dickson D.W. (2019). The neuropathological diagnosis of Alzheimer’s disease. Mol. Neurodegener..

[27] Thal D.R., Tomé S.O. (2022). The central role of tau in Alzheimer’s disease: From neurofibrillary tangle maturation to the induction of cell death. Brain Res. Bull..

[28] Kashyap G., Bapat D., Das D., Gowaikar R., Amritkar R.E., Rangarajan G., Ravindranath V., Ambika G. (2019). Synapse loss and progress of Alzheimer’s disease -A network model. Sci. Rep..

[29] Breijyeh Z., Karaman R. (2020). Comprehensive Review on Alzheimer’s Disease: Causes and Treatment. Molecules.

[30] Butterfield D.A., Halliwell B. (2019). Oxidative stress, dysfunctional glucose metabolism and Alzheimer disease. Nat. Rev. Neurosci..

[31] Tufarelli V., Colonna M.A., Losacco C., Puvača N. (2023). Biological Health Markers Associated with Oxidative Stress in Dairy Cows during Lactation Period. Metabolites.

[32] Militello R., Luti S., Gamberi T., Pellegrino A., Modesti A., Modesti P.A. (2024). Physical Activity and Oxidative Stress in Aging. Antioxidants.

[33] Perry G., Cash A.D., Smith M.A. (2002). Alzheimer Disease and Oxidative Stress. BioMed Res. Int..

[34] Sultana R., Butterfield D.A. (2010). Role of Oxidative Stress in the Progression of Alzheimer’s Disease. J. Alzheimer’s Dis..

[35] Alqahtani T., Deore S.L., Kide A.A., Shende B.A., Sharma R., Chakole R.D., Nemade L.S., Kale N.K., Borah S., Deokar S.S. (2023). Mitochondrial dysfunction and oxidative stress in Alzheimer’s disease, and Parkinson’s disease, Huntington’s disease and Amyotrophic Lateral Sclerosis -An updated review. Mitochondrion.

[36] Dhapola R., Beura S.K., Sharma P., Singh S.K., HariKrishnaReddy D. (2024). Oxidative stress in Alzheimer’s disease: Current knowledge of signaling pathways and therapeutics. Mol. Biol. Rep..

[37] Tramutola A., Lanzillotta C., Perluigi M., Butterfield D.A. (2017). Oxidative stress, protein modification and Alzheimer disease. Brain Res. Bull..

[38] Bai R., Guo J., Ye X.-Y., Xie Y., Xie T. (2022). Oxidative stress: The core pathogenesis and mechanism of Alzheimer’s disease. Ageing Res. Rev..

[39] Myhre O., Utkilen H., Duale N., Brunborg G., Hofer T. (2013). Metal Dyshomeostasis and Inflammation in Alzheimer’s and Parkinson’s Diseases: Possible Impact of Environmental Exposures. Oxidative Med. Cell. Longev..

[40] Greenough M.A., Camakaris J., Bush A.I. (2013). Metal dyshomeostasis and oxidative stress in Alzheimer’s disease. Neurochem. Int..

[41] Wang L., Yin Y.-L., Liu X.-Z., Shen P., Zheng Y.-G., Lan X.-R., Lu C.-B., Wang J.-Z. (2020). Current understanding of metal ions in the pathogenesis of Alzheimer’s disease. Transl. Neurodegener..

[42] Leko M.B., Horvat L.L., Popovački E.Š., Zubčić K., Hof P.R., Šimić G. (2023). Metals in Alzheimer’s Disease. Biomedicines.

[43] Yang G., Liu H., Ma D.-L., Leung C.-H. (2019). Rebalancing metal dyshomeostasis for Alzheimer’s disease therapy. JBIC J. Biol. Inorg. Chem..

[44] Metaxas A. (2021). Imbalances in Copper or Zinc Concentrations Trigger Further Trace Metal Dyshomeostasis in Amyloid-Beta Producing Caenorhabditis elegans. Front. Neurosci..

[45] Chen L., Shen Q., Liu Y., Zhang Y., Sun L., Ma X., Song N., Xie J. (2025). Homeostasis and metabolism of iron and other metal ions in neurodegenerative diseases. Signal Transduct. Target. Ther..

[46] Kenche V.B., Barnham K.J. (2011). Alzheimer’s disease & metals: Therapeutic opportunities. Br. J. Pharmacol..

[47] Santos M.A., Chand K., Chaves S. (2016). Recent progress in multifunctional metal chelators as potential drugs for Alzheimer’s disease. Coord. Chem. Rev..

[48] Zafar R., Zubair M., Ali S., Shahid K., Waseem W., Naureen H., Haider A., Jan M.S., Ullah F., Sirajuddin M. (2021). Zinc metal carboxylates as potential anti-Alzheimer’s candidate: In vitro anticholinesterase, antioxidant and molecular docking studies. J. Biomol. Struct. Dyn..

[49] Cai R., Wang L.-N., Fan J.-J., Geng S.-Q., Liu Y.-M. (2019). New 4-N-phenylaminoquinoline derivatives as antioxidant, metal chelating and cholinesterase inhibitors for Alzheimer’s disease. Bioorg. Chem..

[50] Wang Y., Yang Y., Hong K.H., Ning Y., Yu P., Ren J., Ji M., Cai J. (2019). Design, synthesis and evaluation of a novel metal chelator as multifunctional agents for the treatment of Alzheimer’s disease. Bioorg. Chem..

[51] Xu P., Zhang M., Sheng R., Ma Y. (2017). Synthesis and biological evaluation of deferiprone-resveratrol hybrids as antioxidants, Aβ 1–42 aggregation inhibitors and metal-chelating agents for Alzheimer’s disease. Eur. J. Med. Chem..

[52] Chand K., Alsoghier H.M., Chaves S., Santos M.A. (2016). Tacrine-(hydroxybenzoyl-pyridone) hybrids as potential multifunctional anti-Alzheimer’s agents: AChE inhibition, antioxidant activity and metal chelating capacity. J. Inorg. Biochem..

[53] Manral A., Saini V., Meena P., Tiwari M. (2015). Multifunctional novel Diallyl disulfide (DADS) derivatives with β-amyloid-reducing, cholinergic, antioxidant and metal chelating properties for the treatment of Alzheimer’s disease. Bioorg. Med. Chem..

[54] Wang Z.-M., Xie S.-S., Li X.-M., Wu J.-J., Wang X.-B., Kong L.-Y. (2015). Multifunctional 3-Schiff base-4-hydroxycoumarin derivatives with monoamine oxidase inhibition, anti-β-amyloid aggregation, metal chelation, antioxidant and neuroprotection properties against Alzheimer’s disease. RSC Adv..

[55] Xie S., Chen J., Li X., Su T., Wang Y., Wang Z., Huang L., Li X. (2015). Synthesis and evaluation of selegiline derivatives as monoamine oxidase inhibitor, antioxidant and metal chelator against Alzheimer’s disease. Bioorg. Med. Chem..

[56] Sang Z., Li Y., Qiang X., Xiao G., Liu Q., Tan Z., Deng Y. (2015). Multifunctional scutellarin–rivastigmine hybrids with cholinergic, antioxidant, biometal chelating and neuroprotective properties for the treatment of Alzheimer’s disease. Bioorg. Med. Chem..

[57] Wu M.-Y., Esteban G., Brogi S., Shionoya M., Wang L., Campiani G., Unzeta M., Inokuchi T., Butini S., Marco-Contelles J. (2016). Donepezil-like multifunctional agents: Design, synthesis, molecular modeling and biological evaluation. Eur. J. Med. Chem..

